# Pharmacological Treatment of Painful HIV-Associated Sensory Neuropathy: A Systematic Review and Meta-Analysis of Randomised Controlled Trials

**DOI:** 10.1371/journal.pone.0014433

**Published:** 2010-12-28

**Authors:** Tudor J. C. Phillips, Catherine L. Cherry, Sarah Cox, Sarah J. Marshall, Andrew S. C. Rice

**Affiliations:** 1 Department of Anaesthetics, Pain Medicine and Intensive Care, Imperial College London, Chelsea and Westminster Hospital Campus, London, United Kingdom; 2 Centre for Virology, Burnet Institute, Melbourne, Australia; 3 Infectious Diseases Unit, Alfred Hospital, Melbourne, Australia; 4 Department of Medicine, Monash University, Melbourne, Australia; 5 Chelsea and Westminster NHS Foundation Trust, London, United Kingdom; 6 East Kent Hospitals University Foundation Trust and Pilgrims Hospices, Kent, United Kingdom; McGill University Health Center, Canada

## Abstract

**Background:**

Significant pain from HIV-associated sensory neuropathy (HIV-SN) affects ∼40% of HIV infected individuals treated with antiretroviral therapy (ART). The prevalence of HIV-SN has increased despite the more widespread use of ART. With the global HIV prevalence estimated at 33 million, and with infected individuals gaining increased access to ART, painful HIV-SN represents a large and expanding world health problem. There is an urgent need to develop effective pain management strategies for this condition.

**Method and Findings:**

Objective: To evaluate the clinical effectiveness of analgesics in treating painful HIV-SN. Design: Systematic review and meta-analysis. Data sources: Medline, Cochrane central register of controlled trials, www.clinicaltrials.gov, www.controlled-trials.com and the reference lists of retrieved articles. Selection criteria: Prospective, double-blinded, randomised controlled trials (RCTs) investigating the pharmacological treatment of painful HIV-SN with sufficient quality assessed using a modified Jadad scoring method. Review methods: Four authors assessed the eligibility of articles for inclusion. Agreement of inclusion was reached by consensus and arbitration. Two authors conducted data extraction and analysis. Dichotomous outcome measures (≥30% and ≥50% pain reduction) were sought from RCTs reporting interventions with statistically significant efficacies greater than placebo. These data were used to calculate RR and NNT values.

**Results:**

Of 44 studies identified, 19 were RCTs. Of these, 14 fulfilled the inclusion criteria. Interventions demonstrating greater efficacy than placebo were smoked cannabis NNT 3.38 95%CI(1.38 to 4.10), topical capsaicin 8%, and recombinant human nerve growth factor (rhNGF). No superiority over placebo was reported in RCTs that examined amitriptyline (100mg/day), gabapentin (2.4g/day), pregabalin (1200mg/day), prosaptide (16mg/day), peptide-T (6mg/day), acetyl-L-carnitine (1g/day), mexilitine (600mg/day), lamotrigine (600mg/day) and topical capsaicin (0.075% q.d.s.).

**Conclusions:**

Evidence of efficacy exists only for capsaicin 8%, smoked cannabis and rhNGF. However,rhNGF is clinically unavailable and smoked cannabis cannot be recommended as routine therapy. Evaluation of novel management strategies for painful HIV-SN is urgently needed.

## Introduction

HIV-associated distal sensory neuropathy (HIV-SN) is a frequently occurring neurological complication of HIV infection. HIV-SN prevalence has increased despite (or because of) the introduction of otherwise successful antiretroviral therapy [1]. HIV-SN is one of the most prevalent problems experienced by people receiving antiretroviral therapy and the associated pain has a major impact on quality of life in otherwise largely healthy individuals. HIV-SN is a distal symmetrical axonal, predominantly sensory polyneuropathy that affects the feet and less frequently the hands. HIV-SN is comprised of at least two clinically indistinguishable, and often coexisting, neuropathies: A distal sensory polyneuropathy associated with HIV disease itself (HIV-DSP), and a distal sensory polyneuropathy associated with antiretroviral treatment, Antiretroviral toxic neuropathy (HIV-ATN). HIV-DSP was recognised early in the HIV pandemic [2] and is associated with advanced HIV disease [1] [3]. HIV-ATN was initially observed following the introduction of particular nucleoside reverse transcriptase inhibitors (NRTI) – stavudine, didanosine and zalcitabine - the ‘dNRTIs’ [4]–[5]. The presence of sensory neuropathic symptoms in an ARV naïve patient is highly suggestive of HIV -DSP. Often only a temporal association between the onset of symptoms and the starting of a particular ARV agent gives the only hint as to aetiology, as in most other clinical respects the two are almost identical.

The introduction of combination antiretroviral therapy (cART) in the mid 1990s dramatically reduced the morbidity and mortality associated with HIV among patients who have access to treatment [6]. Life expectancy with HIV in well-resourced countries is now estimated to be up to two-thirds that of the general population [7]–[8]. While the incidence of most neurological complications of HIV has fallen with the introduction of effective therapy, rates of HIV-SN have been rising since the first effective antiretroviral drugs were developed [9]. Recent estimates of HIV-SN prevalence among cohorts with access to cART range from 20% [10] to >50% [11]. Importantly, the available evidence suggests that HIV-SN prevalence remains high among cART-treated patients, even in countries where known neurotoxic antiretroviral drugs such as stavudine are no longer commonly used. Depending on the population surveyed, HIV-SN, regardless of previous ARV exposure, has a prevalence of between 13% [12] and >50% [13]–[14] of HIV infected individuals, of whom 40% experience severe pain, ≥5/10 Numeric Pain Rating Scale (NPRS), and 90% experiencing some pain, which can be severely debilitating [1]. In less well-resourced centres, use of stavudine, an inexpensive and effective antiretroviral, in first-line HIV treatment remains common despite the high risk of neurotoxicity [15].

Importantly two recent studies have emphasised the continued and growing global impact of HIV-SN. A large cross-sectional study of 598 HIV infected individuals in South Africa, reported that the frequency of symptomatic HIV-SN increases from 23% to 40% following exposure to ART therapy, with 60% being symptomatic if previously exposed to stavudine [16]. Another large cross-sectional study from the US studying 1539 HIV infected individuals has reported that 57%(881) demonstrated evidence of the presence of HIV-SN, with 38% of these individuals reporting pain [17].

Current estimates of global HIV prevalence stand at 33 million, with 2.7 new infections each year and more patients gaining access to cART [15]. With high rates of HIV-SN now reported globally, and up to 90% of affected patients experiencing potentially debilitating neuropathic pain, HIV-SN represents a large and potentially worsening source of global HIV-related morbidity. There is an urgent need to understand better the pathogenesis of HIV-SN, to identify risk factors, and identify and implement effective preventative and pain management strategies.

Evidence-based guidelines for the pharmacological management of neuropathic pain tend to focus on a “blanket” approach of recommending therapies across the spectrum of neuropathic pain, irrespective of the underlying condition [18]–[19]. Recent NICE guidance for the management of neuropathic pain in “non-specialist settings” have adopted this approach [20]. This may not be appropriate for HIV-SN for three main reasons. Firstly, neuropathic pain is a heterogeneous phenomenon, both within and across underlying conditions, and evidence obtained from the study of an analgesic in one condition cannot necessarily be applied to another [21]–[22]. Secondly, in high, middle and low income countries the pain associated with HIV-SN will usually be managed outside of specialist pain management clinics, so appropriate, disease specific guidance may be required. Finally, there are a number of randomised controlled trials (RCTs) conducted in HIV-SN, which were not identified in the NICE guidance. Therefore, we have conducted a systematic review and meta-analysis to elucidate the evidence base for pharmacological management of neuropathic pain in HIV-SN.

## Methods

### Eligibility, data sources and search strategy

In accordance with PRISMA [23], we sought to identify RCTs that included patients with painful HIV-SN and reported at least one clinically relevant pain outcome measure.

A systematic search, without language restrictions, was conducted on 20 June 2008, and a follow-up search on 22 February 2010, with the following databases: Medline (from 1966 to date searched), The Cochrane central register of controlled trials (Cochrane Library 2010, Issue 2), www.clinicaltrials.gov (a US registry of clinical trials) and www.controlled-trials.com (a meta-registry of controlled trials). Search terms used were: “HIV” “AIDS” “pain” “painful” “neuropathy” “neuropathic”, in combination with “random” “randomised” and “double-blinded”. Further trials were identified by hand searching the reference lists of identified trials and review articles, relevant NICE guidelines and Health Technology Assessment reports.

### Study selection and risk of bias assessment

We excluded animal studies, reviews, letters, abstract-only trials, open-label trials, and trials that were not randomised. The identified RCTs then underwent independent quality assessment by four authors (TJCP, CLC, SC and ASCR) using a 7-point modified “Jadad” scoring system that assessed the presence and quality of double-blinding, randomisation, study size and reporting of withdrawal and drop outs [24]–[25]. RCTs with a score of less than five points and studies that enrolled fewer than five HIV-SN patients were excluded from the systematic review. Scoring discrepancies between authors were resolved through discussion and consensus; with final arbitration by ASCR.

### Data extraction

Data were extracted from eligible RCTs by one author (TJCP). Data extracted included: year of publication; study design and duration; study sample population and characteristics; withdrawals; interventions; doses; pain and non-pain related primary and secondary outcome measures; and adverse events.

Where possible, dichotomous pain improvement outcome data were extracted from RCTs that reported efficacy superior to placebo. Intention to treat (ITT) responder rates for 30% and 50% pain relief were sought for the longest follow-up period reported in each study. If required, authors were contacted for missing or unreported data.

RCTs in which the primary pain outcome of a studied intervention did not show efficacy greater than placebo in the intention to treat population, were not included in subsequent analyses.

### Statistical analysis

For each intervention the extracted dichotomous outcomes were used to calculate numbers needed to treat (NNT) by two authors (TJCP and ASCR), with 95% confidence intervals for 30% and 50% pain improvement responders. We originally planned to access heterogeneity according to the method of Armitage & Berry [26], and visually [27] however as only three studies were used in the meta-analysis, this was felt to be inappropriate. Similarly, a sensitivity analysis was not performed, as there were insufficient data. All calculations were undertaken using Microsoft Excel 2007.

## Results

**Figure 1:**
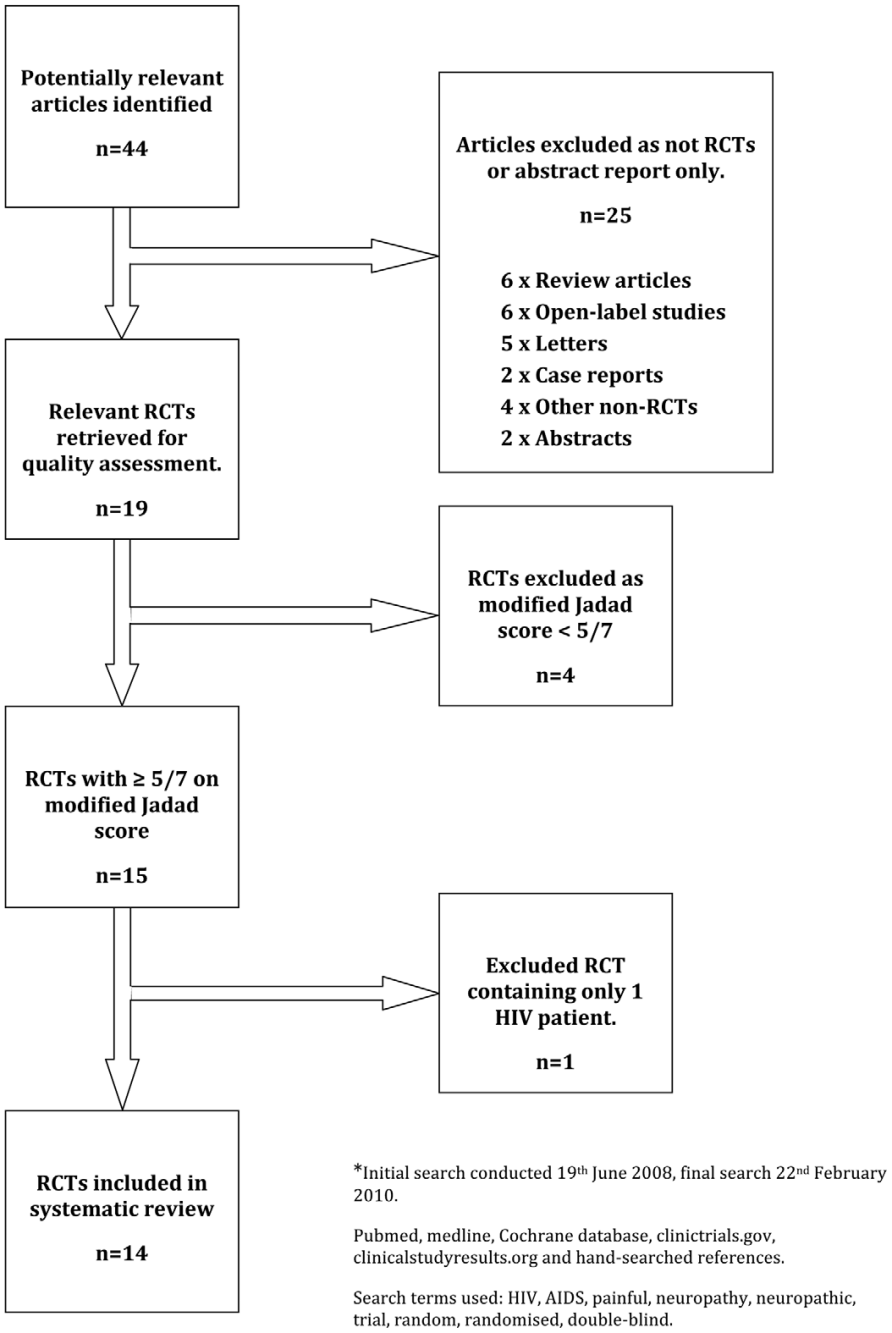
PRISMA flow diagram of included randomized controlled trials.

We identified 44 potentially relevant articles (Figure 1). Twenty-five articles were excluded after screening identified these as being a review article, letter, open-label study, case report or other non-RCT study. The remaining 19 RCTs were retrieved and independently reviewed by four authors (TJCP, CLC, SC and ASCR). Four articles were excluded at this stage by scoring <5 out of 7-points with the modified Jadad score. A further RCT was excluded as having <5 HIV-SN patients enrolled. Details of these excluded RCTs, and therefore of interventions that must be regarded as not having been adequately tested, are shown in Table 1.

**Table 1:** Studies excluded from the analysis.

Reference	Treatment	Primary Reason for Exclusion
[37]	Acetyl-L-carnitine	Review
[61]	Acetyl-L-carnitine	Review
[43]	Cannabinoids	Review
[62]	Lamotrigine	Review
[63]	Antidepressants	Review
[64]	Herbal medicine	Review
[44]	8% capsaicin patch	Open-label
[45]	8% capsaicin patch	Abstract
[33]	Acetyl-L-carnitine	Open-label
[34]	Acetyl-L-carnitine	Open-label
[65]	Recombinant human NGF	Open-label
[66]	Flecainide	Open-label
[67]	5% lidocaine patch	Open-label
[68]	Acupuncture	Letter
[69]	Acupuncture	Letter
[70]	Acupuncture	Letter
[46]	Gabapentin	Letter
[47]	Gabapentin	Letter
[49]	Gabapentin	Case report
[71]	Prednisolone	Case report
[48]	Gabapentin	Abstract
[42]	Smoked cannabis	Other non-RCT
[35]	Acetyl-L-carnitine	Other non-RCT
[72]	Acupuncture	Other non-RCT
[32]	Acetyl-L-carnitine	Other non-RCT
[73]	5% lidocaine patch	Modified Jadad score <5
[74]	Mexiletine	Modified Jadad score <5
[75]	Memantine	Modified Jadad score <5
[76]	Nimodipine	Modified Jadad score <5
[52]	Lamotrigine	<5 patients enrolled

The remaining 14 RCTs were retained for further analysis (Table 2). Of the 14 trials retained for further analysis, 13 were of a parallel group design and one a cross-over design. All were placebo controlled with one using “active” placebo [28]. Data extraction was for the longest follow-up period reported by the article. In most cases this was to the end of the treatment phase, except for a study of a topical 8% capsaicin [28] that reported data for 12 weeks after a single treatment application.

**Table 2:** Characteristics and results of included studies.

Reference	Participants recruited (completed)	Design and duration	Intervention (n = patient episodes)	Maximum dose studied	Primary Outcome	Data (ITT)	Superior to placebo?
Youle M et al 2007 [36]	90(76)	Parallel: 2 wks	Acetyl-L-carnitine 500 mg bd i.m. (n = 43); placebo (n = 47)	1000mg/day	VAS (0–10cm) change: baseline to wk 2.	ITT: Acetyl-L-carnitine: −1.32 (SD 1.84); placebo −0.61 (SD 1.55)(p = 0.07)	No
Shlay JC et al 1998 [38]	136 (105)	Parallel: 14 wks	Amitriptyline (n = 71); placebo (n = 65)	75mg/day	GP score: change baseline to wk 14.	ITT with LOCF: Amitriptyline: −0.26; placebo: −0.30;difference 0.00 95%CI(−0.18 to 0.19)(p = 0.99)	No
Paice JA et al 2000 [29]	26(14)	Parallel:4 wks.	Capsaicin 0.075% cream q.d.s. (n = 15); placebo (n = 11)	0.075% q.d.s.	NRS (0–10): change from baseline to wk 4.	No numeric data given. Stated no statistically significant difference between capsaicin 0.075% and placebo (p>0.05)	No
Simpson DM et al 2008 [28]	307(274)	Parallel: 12 wks follow-up.	Capsaicin 8% patch for 30min (n = 72); 60min (n = 78); 90min (n = 75); placebo (capsaicin 0.04%) (n = 82)	8% for 90min.	NPRS: % change baseline to wk 12.	ITT with LOCF: Capsaicin: −22.8 (SD 30.6); placebo −10.7 (SD 30.8); (p = 0.0026)	Yes
Abrams DI et al 2007 [40]	55(50)	Parallel: 5 days	Smoked cannabis (n = 27); placebo (n = 28)	3.56% Δ-9- tetrahydrocannabinol t.d.s.	VAS: % change from baseline to day 5.	ITT: Cannabis: −34% (IQR −71 to −16); placebo −17% (IQR – 29 to 8) (p = 0.03)	Yes
Ellis RJ et al 2009 [41]	34(27)	Crossover: 5 days, 2 wks washout, 5 days treatment.	Smoked cannabis (n = 28); placebo (n = 28)	Max tolerable: 1 to 8% Δ-9- tetrahydrocannabinol q.d.s.	DDS (0–20): median change from baseline to day 5.	Difference in DDS reduction cannabis vs placebo for PP: −3.3 p = 0.016, no data for ITT: said to be ‘similar’ with p = 0.020	Yes
Hahn K et al 2004 [30]	26(24)	Parallel: 4 wks treatment.	Gabapentin (n = 15); placebo (n = 11)	2400mg/day	VAS: median change: baseline to wk 4.	Gabapentin: −44.1; placebo: −29.8. Stated as being not statistically significant.	No
Simpson DM et al 2010 [50]	302(241)	Parallel: 14 wks treatment.	Pregabalin (n = 151); placebo (n = 151)	1200mg/day	NPRS: mean change: baseline to wk 14.	ITT: Pregabalin: −2.88; placebo −2.63 (p = 0.39)	No
Simpson DM et al 2000 [51]	42(29)	Parallel: 14 wks treatment	Lamotrigine (n = 20); placebo (n = 22)	300mg/day	GP score: mean change: baseline to wk 14.	ITT with LOCF: Lamotrigine: −0.242 (SE 0.092); placebo: −0.183 (SE 0.087) (p = 0.65)	No
Simpson et al 2003 [31]	227(172)	Parallel:12 wks treatment.	Lamotrigine (n = 150); placebo (n = 77)	600mg/day	GP score: change: baseline to wk 12.	PP: Lamotrigine vs placebo. No data given, stated no statistically significant difference seen in all or ARV stratum.	No
Keiburtz K et al 1998 [39]	145(104)	Parallel: 8 wks	Mexilitine (n = 48); amitriptyline (n = 47); placebo (n = 50)	Mexilitine: 300mg/day Amitriptyline: 100mg/day	GP score: mean change: baseline to wk 8.	ITT: Amitriptyline: −0.31 (SD 0.31); mexilitine: −0.23 (SD 0.41); placebo −0.20 (SD 0.30) No p value given, stated no statistical significance	No
McArthur JC et al 2000 [53]	270(235)	Parallel:18 wks	Recombinant human NGF (n = 180); placebo (n = 90)	0.3µg/kg s.c. twice weekly	GP score: median change: baseline to wk 18.	ITT with LOCF: NGF 0.1µg/kg: −0.18 (−0.10 to −0.25)(p = 0.05); NGF 0.3µg/kg: −0.21 (−0.14 to −0.29)(p = 0.04); placebo: 0.06 (+0.01 to −0.14)	Yes
Simpson DM et al 1996 [54]	104(81)	Parallel: 12 wks	Peptide-T (n = 40); placebo (n = 41)* PP data	6mg/day intranasal	Modified GP score: mean change: baseline to wk 12.	PP: Peptide-T: −0.24 (±0.45); placebo −0.39 (±0.19) (p = 0.32). ITT results not presented but stated showed the ‘same pattern’.	No

GP - Gracely Pain Score, VAS – Visual Analogue Scale, ITT – Intention To Treat population, PP -Per Protocol population, NRS – Numerical Rating Scale, NPRS- Numerical Pain Rating Scale, DDS – Descriptor Differential Scale, LOCF - Last Observation Carried Forward.

In two studies [29] and [30] no reference to ITT analysis was made. In one of these RCTs studying topical capsaicin 0.075% efficacy [29] no primary outcome data were published, as it was reported that no superiority to placebo was seen. In a study of lamotrigine efficacy [31] only a per protocol (PP) population data analysis was undertaken. This was reported to show no superiority over placebo; however no primary outcome data were reported.

Of the four trials that reported superiority of an intervention over placebo, three reported dichotomous pain outcome measures. Where possible we used responder rate data for ≥30% and ≥50% improvement in pain as measured using Visual Analogue Scale (VAS) or Numerical Pain Rating Scale (NPRS). These data were requested from the authors if they had not been reported.

### Acetyl –L-carnitine

Whilst acetyl-L-carnitine has been the subject of six articles [32]–[37] in the treatment of painful HIV-SN, only one was an RCT [36] and eligible for inclusion. This was a parallel group trial of acetyl-L-carnitine (1000mg/day) and placebo intramuscular injections. In this RCT acetyl-L-carnitine, in an analysis of the PP population, showed a modest superiority to placebo. However an analysis of the ITT population did not show superiority to placebo: mean change in VAS (0–10cm)(SD) from baseline to the end of week 2: acetyl-L-carnitine −1.32 (1.84); placebo −0.61 (1.55) p = 0.07. Consequently we undertook no further analysis of this trial.

### Amitriptyline and Mexilitine

Two trials [38] and [39] that were included studied the efficacy of amitriptyline. Both trials compared amitriptyline to placebo and another intervention. One RCT [38] examined efficacy of amitriptyline as part of a trial also assessing acupuncture treatment. However despite being described as a parallel group, placebo controlled RCT, its design was complex. Consequently the results of this trial are difficult to evaluate. In particular bias may have been introduced because of unconventional randomisation procedures and because true placebo controls were not used. Specifically, patients were allowed to ‘opt-out’ of being randomised to the amitriptyline arms of the trial based on personal preference. In addition, many participants included in the analysis of amitriptyline efficacy, had also received acupuncture or sham acupuncture, further complicating analysis. Ignoring the methodological concerns, amitriptyline demonstrated no superiority to placebo in the primary outcome measure. The mean change in Gracely pain scores from baseline to week 14 was −0.26 with amitriptyline (maximum dose 75mg/day) and −0.30 with placebo. The difference between amitriptyline and placebo was: 0.00 95%CI(−0.18 to 0.19) p = 0.99.

The second trial [39] compared amitriptyline, mexilitine and placebo. This trial was terminated early following an interim review of results. It was deemed by the trial monitoring board that further enrolment into the study was unlikely to detect significant differences in either amitriptyline or mexilitine arms compared to placebo. No superiority was reported in reducing mean Gracely pain scores (SD) from baseline to the end of treatment week 8 for: amitriptyline (maximum dose 100mg/day) −0.31 (0.31); mexilitine −0.23 (0.41); compared to placebo −0.20 (0.30).

### Smoked Cannabis

The original literature search found four articles related to cannabinoid use and painful HIV-SN. Only two were RCTs [40]–[41]. The excluded articles included one clinical survey [42] and one review article [43].

One of these included articles [41] was a cross-over study that compared the efficacy of smoked cannabis (maximum tolerated dose 1 to 8% Δ-9-tetrahydrocannabinol q.d.s.) to placebo cigarettes in reducing subjects pain measured using the Descriptor Differential Scale (DDS). The DDS is a ratio scale (0 to 20) containing 24 words describing pain intensity and unpleasantness. Smoked cannabis was reported to be superior to placebo in reducing DDS from baseline to end of treatment day five in the PP population. The median difference between cannabis and placebo was −3.3 out of 20; p = 0.016. No data were reported for the ITT analysis, however the authors stated that the PP analysis was similar to the ITT analysis with p = 0.02. VAS data not reported by the authors, but was supplied on request, relating to cannabis and placebo subjects who reported a ≥30% (18/34 and 7/34 respectively) and ≥50% (13/34 and 4/34 respectively) improvement in pain intensity.

This trial reported a high proportion of inadvertent unblinding amongst subjects following dose titration with smoked cannabis cigarettes in the treatment arms, but not with placebo cigarettes.

A second study [40] compared smoked cannabis (3.56% Δ-9-tetrahydrocannabinol t.d.s.) to placebo cigarettes in a parallel group RCT. Smoked cannabis was shown to be superior to placebo in reducing pain from baseline to end of treatment day 5 in the ITT analysis: cannabis −34% (IQR −71 to −16), placebo −17% (IQR −29 to 8) p = 0.03. More subjects reported ≥30% VAS improvement with smoked cannabis compared to the placebo: 13/27 and 6/27 respectively.

Inclusion into the study required subjects to have had previous exposure to cannabis, with current users asked to discontinue prior to the study. Of note no attempt was made to assess unintentional unblinding during the course of the study, which may have been high due to subjects' previous experience with smoked cannabis.

Using the ITT analysis dichotomous VAS data from both trials, an NNT for smoked cannabis was calculated as 3.38 95%CI (2.19 to 7.50) (Table 3)

**Table 3:** Summary of RCTs which demonstrated treatment superior to placebo, for which Relative Risk and Number Needed to Treat values could be calculated.

	Active Treatment (maximum tested dose)	Number of patient Episodes	Benefit Efficacy on Treatment (≥30% improvement VAS	Efficacy on Placebo (≥30% improvement VAS)	RR (95% CI)	NNT (95% CI)
Smoked cannabis						
Abrams et al 2007 [40]	Smoked cannabis: 3.56% Δ-9-tetrahydrocannabinol	55 (50)	13/27	6/28	2.17 (0.97 to 4.86)	3.86 (1.98 to 71.11)
Ellis et al 2009 [41]	Smoked cannabis: 8% Δ-9-tetrahydrocannabinol	68 (56)	18/34	7/34	2.57 (1.24 to 5.35)	3.09 (1.98 to 9.30)
Abrams et al [40]+Ellis et al [41]	Combined smoked cannabis studies	122 (106)	31/61	15/61	2.38 (1.38 to 4.10)	3.38 (2.19 to 7.50)

### Topical Capsaicin

Four trials [44] [29] [28] and [45] were found that assessed topical capsaicin efficacy in painful HIV-SN. Two reports were excluded from further analysis; one was an open-label study [28] and the other has been reported in abstract form only [45]. Of the included trials, one [29] examined the efficacy of topical capsaicin 0.075% cream in a parallel group RCT. The authors stated that no superiority of capsaicin 0.075% over placebo in mean improvement in a numeric rating score (NRS) (0–10) was seen, however only graphical data were presented.

A second study [28] examined topical capsaicin 8%. Patients received either the 8% patch or an active placebo (capsaicin 0.04%) in a single application lasting either 30, 60 or 90 minutes. Following this single application patients were followed-up for 12 weeks. Capsaicin 8% was found to be superior to placebo in the percentage reduction of the NPRS (SD) from baseline to week 2 to 12: 8% capsaicin: −22.8 (30.6); compared to placebo: −10.7 (30.8), (p = 0.0026). The study also reported responder rates as percentage of patients measured on the NPRS who experienced ≥30% mean reduction in pain: capsaicin 8%: 76/225; placebo (capsaicin 0.04%): 15/82; p = 0.0092. It is not possible to calculate an NNT that is strictly comparable to those calculated for other studies included in this review since the placebo control used here was not pharmacologically inactive. However, as an informative exercise using these data, and presuming that the control capsaicin 0.04% is a true placebo, an NNT of 6.46 95%CI(3.86–19.69) was calculated for treatment with capsaicin 8% patch.

### Gabapentin

Only one retrieved report related to treatment of painful HIV-SN with gabapentin was an RCT. Four additional articles were excluded. Two were letters [46]–[47] one an abstract [48], and one a case series [49]. The included study [30] compared gabapentin (titrated to a maximum of 2400mg/day) to placebo in a parallel group RCT. At the longest treatment period assessed, no difference in efficacy was reported between gabapentin and placebo groups for the primary outcome measure, median change in VAS (0–100mm) baseline to end of week 4: gabapentin: −44.1, placebo: −29.8. No indication of variance or p value was documented.

It is noteworthy that this trial demonstrated an unusual placebo response. The placebo subjects' pain VAS baseline remained unchanged for the first two weeks, after which a stronger placebo response followed to week 4. This unusual placebo response may have contributed to the apparent superiority of gabapentin over placebo at week 2, which was not evident at week 4.

### Pregabalin

One large multi-centre RCT [50] examined the efficacy of pregabalin, titrated over 2 weeks to a maximum tolerated dose up to 1200mg/day, in a multicentre, 14 week parallel group, placebo controlled RCT. No superiority of pregabalin over placebo in the primary pain outcome measure was reported: mean change in NPRS baseline to end of week 14: pregabalin −2.88; placebo −2.63, p = 0.39.

### Lamotrigine

Three trials assessing the efficacy of lamotrigine in painful HIV-SN were identified [51], [52] and [29], [31]). One trial [52], enrolled only one painful HIV-SN patient (to the placebo control group) and was therefore excluded from further analysis. The included lamotrigine trials [51] and [31] were both conducted by the same group; with [51] being smaller and preceding [31]. The smaller study [51] did demonstrate some efficacy superior to placebo when the primary outcome for the PP population was analysed. However in the ITT analysis with ‘last value carried forward’ (LVCF), lamotrigine was not superior to placebo: improvement in mean Gracely pain score (SE): lamotrigine: −0.242 (0.009); placebo: −0.183 (0.087); (p = 0.65). The large number of drop-outs in the lamotrigine group (n = 11 of 20) compared to placebo (n = 3 of 22) suggest a narrow therapeutic index and make interpretation of the trial results difficult.

Similarly the larger trial [31], where participants were stratified according to previous exposure to neurotoxic ARVs, did not demonstrate a superiority of lamotrigine over placebo for the primary outcome measure (mean improvement in Gracely pain score) in the total cohort or in either stratum. However lamotrigine did show superiority to placebo in the neurotoxic ARV-exposed stratum in a secondary outcome measure, mean improvement in VAS (0–100mm) baseline to end of treatment: lamotrigine: −27.1; compared to placebo: −9.0; p = 0.003.

For each stratum the number of responders (≥30% improvement in VAS) were calculated from the published data. For the neurotoxic ARV stratum: lamotrigine 36/62, placebo 7/30 (p = 0.02) and for no exposure to neurotoxic ART: lamotrigine 46/88, placebo 21/47. As an informative exercise using these data the NNT for lamotrigine was calculated for each stratum, and for the overall trial. Subjects with exposure to neurotoxic ARVs: 2.88 95%CI(1.84 to 6.57); no exposure to neurotoxic ARVs: 13.17 95%CI(3.96 to −9.95) and for the unstratified population: 6.09 95%CI(3.51 to 23.08)(Not included in Table 3 as no superiority of lamotrigine over placebo was demonstrated for any primary endpoint).

### NGF

One RCT [53] examined the efficacy of subcutaneous recombinant human Nerve Growth Factor (rhNGF) in the treatment of painful HIV-SN. This study assessed two doses (0.1 and 0.3µg/kg) given twice weekly compared with placebo for 18 weeks. rhNGF was superior to placebo for the primary outcome measure in the ITT analysis; median change of the Gracely pain score from baseline to end of week 18: rhNGF 0.1µg/kg: −0.18 (−0.10 to −0.25) p = 0.05, 0.3µg/kg: −0.21 (−0.14 to −0.29) p = 0.04, and placebo: 0.06 (+0.01 to −0.14).

No significant dose effect was reported and no differential effect was seen based on baseline stratification of subjects according to neurotoxic ARV drug exposure. As rhNGF was reported to be associated with myalgia, there may have been inadvertent breaking of the blinding.

Dichotomous data were requested from the authors however we were unable to calculate RR and NNT values for rhNGF from the data provided.

### Prosaptide and Peptide –T

Two trials [54], [55] examined the efficacy of the novel agents in placebo controlled parallel group RCTs. One [55] reported the use of subcutaneous prosaptide (maximum dose of 16mg/day) over 6 treatment weeks and did not report efficacy superior to placebo in the primary outcome measure; mean change in Gracely pain score baseline to week 6. The study was terminated after a planned interim futility analysis. Another trial [54] studied efficacy of intranasal peptide T (maximum dose 6mg/day), over 12 treatment weeks, but reported no superiority over placebo in the primary outcome measure; mean change in a modified Gracely pain score baseline to end of week 12.

## Discussion

This systematic review found that RCT evidence of analgesic efficacy superior to placebo in the context of HIV-SN pain exists only for smoked cannabis, rhNGF and high dose (8%) topical capsaicin. Several other agents have been examined in high quality RCTs and found to be no more effective than placebo for managing HIV-SN pain in the doses examined, specifically acetyl-L carnitine (1g/day), amitriptyline (100mg/day), topical capsaicin 0.075%, gabapentin (2.4g/day), mexilitine (600mg/day), peptide –T (6mg/day), pregabalin(1200mg/day), lamotrigine (600mg/day) and prosaptide (16mg/day). Therefore, there is evidence that both of the first line therapies (pregabalin and amitriptyline) recommended in the NICE guidance for non-specialist management of neuropathic pain show no superiority to placebo in the management of pain in HIV-SN [20].

Of the pharmacological interventions shown to be effective for HIV-SN in RCTs, only topical capsaicin 8% is currently approved for marketing for neuropathic pain indications. In Europe 8% capsaicin has been approved for the treatment of peripheral neuropathic pain in non-diabetic adults, whilst the U.S. Food and Drug Administration (FDA) has approved its use only for the indication of post herpetic neuralgia. However, it should also be borne in mind that we located a preliminary report (conference abstract only and therefore excluded from the systematic review) of another parallel group RCT which included 494 patients with HIV-SN in which topical 8% capsaicin was compared to 0.04% topical capsaicin [45]. No analgesic superiority of 8% capsaicin over 0.04% was demonstrated. rhNGF therapy is not currently clinically available and both legal and mental health issues preclude routine recommendation of long term smoked cannabis for pain management [56].

This systematic review represents a comprehensive review of the literature relating to the pharmacological management of painful HIV-SN. It used a predefined protocol for the initial literature search, data extraction and analysis. There was also strict adherence to inclusion quality criteria as assessed by four independent authors using the modified Jadad score, a tool that assesses each study for potential bias as well as evaluating study power.

This systematic review was limited by the paucity of high quality RCTs examining pharmacological treatment of painful HIV-SN. Additionally the heterogeneity of the included studies design and size made evaluation and comparison of trials difficult. In particular, use of the Gracely pain scale (GPS) in five of the 15 included RCTs made evaluation and inter-study comparison complicated. The GPS is a log unit pain outcome measure that is not a frequently used measure outside trials of HIV-SN. In a recent consensus statement regarding core chronic pain outcome measures [57] it was not one of the recommended pain scales. Several of the studies utilising the Gracely pain score also included more validated secondary pain outcome measures such as either a VAS score or a NPRS. These were used here in preference to the Gracely pain score in the calculation of NNT and RR.

The Jadad tool has been validated and used widely to identify common and major sources of experimental bias in RCTs identified in systematic reviews. Nevertheless, whilst the use of the modified Jadad score improves the probability that only high quality RCTs were included in the systematic review, its use may conceivably have biased our systematic review in favour of more recently tested agents. The RCTs associated with these agents now routinely report the information required by the modified Jadad tool, because of the nature of the evolution of RCT methodology over the past few years.

Both of the RCTs that examined the efficacy of smoked cannabis, were of high quality, however the apparent marked superiority of smoked cannabis to placebo cigarettes should be tempered by the high proportion of potential unblinding measured in [41] (92% correctly guessing treatment allocation after treatment crossover), and its lack of measurement in [40] despite participants having all had previous experience of smoked cannabis. In a similar manner, the RCT investigating recombinant human NGF demonstrated a high degree of unblinding related to injection site myalgia, which when accounted for in a separate analysis reported a more attenuated treatment-related difference which consequently lost statistical significance.

Lamotrigine was the subject of two high quality RCTs. Both failed to show superiority over placebo in the primary pain outcome measure, improvement in the GPS in the ITT population. However, in the larger of the two RCTs, analysis of a secondary pain outcome measure, mean improvement in VAS, did demonstrate efficacy superior to placebo in the subpopulation of subjects who had been previously exposed to neurotoxic ARTs. If this stratum alone is examined an NNT of 2.88 is calculated.

Most of the included RCTs did not stratify subjects with painful HIV-SN according to their exposure to neurotoxic ARTs. This stratification was instrumental in demonstrating an efficacy of lamotrigine in neurotoxic ART exposed painful HIV-SN subjects. It is possible to speculate that a similar strategy of stratifying other RCTs might have elucidated other agents with sub-group efficacy, despite lack of observed analgesic efficacy in an unstratified painful HIV-SN subject population. Additionally, the included RCTs were not uniform in their approach to the use of concomitant analgesics; whilst most allowed continued use of drugs at stable doses, two elected to stop them [30] [58]. The use of such concomitant analgesics, and also the inclusion of participants with previously failed therapies, may conceivably have influenced the outcomes of these RCTs.

Gabapentin and pregabalin were the subject of two high quality RCTs in which neither agent was shown to be superior to placebo. This contrasts with the efficacy of these agents demonstrated in other peripheral neuropathic pain conditions [20] [59] [18], [19]. However the gabapentin study was small, with only 30 patients randomised [30]. This finding may therefore represent a ‘failed trial’ rather than a true lack of efficacy.

Amitriptyline efficacy was examined in two large RCTs. The evaluation of one study [38] was made difficult by a complicated study design that may have not been truly randomised or placebo controlled. However the finding that amitriptyline did not display superior analgesic efficacy than placebo in the context of HIV-SN is supported by a similar finding a second, higher quality RCT [39]. Again, this finding directly contrasts with evidence of efficacy for tricyclic antidepressants in a range of other peripheral neuropathic pain conditions [20] [59] [19] [18].

Capsaicin 0.075% cream was the subject of a small RCT enrolling only 26 subjects. The authors stated that capsaicin 0.075% did not demonstrate statistically significant superiority to placebo in the primary pain outcome measure. However, outcome data were published only in a graphical representation of mean current pain scores from baseline to the end of treatment. From this graph there does appear to be a trend for capsaicin to be superior to placebo at this final time point measured at week 4. However a high drop-out rate in both arms resulted in only 6/11 patients remaining in the capsaicin group, and only 8/15 in the placebo group. It is therefore difficult to determine from this study if capsaicin 0.075% was indeed without efficacy. This has two implications: the first being that capsaicin 0.075% might have some degree of clinically relevant efficacy in painful HIV-SN; and secondly, if capsaicin 0.075% is indeed efficacious, then the use of a similar concentration (capsaicin 0.04%) as an active placebo in the large capsaicin 8% patch RCT would change the design of this study from a placebo controlled to a superiority approach.

In the treatment of painful HIV-SN, the lack of efficacy compared with placebo of many agents with proven efficacy in other forms of neuropathic pain has implications in the understanding of neuropathic pain in general. These findings further support the hypothesis that neuropathic pain cannot be considered as a single symptom with a single pathogenesis [21], [22]. A more mechanistic approach to the treatment of specific types of neuropathic pain is therefore warranted as has been established in trigeminal neuralgia and post herpetic neuralgia. Equally, caution should be exercised in the use of neuropathic pain treatment algorithms that do not consider these potential mechanistic differences, as their rationale may be fundamentally flawed.

The absence of studies examining the efficacy of opioid use in painful HIV-SN is notable and mandates additional research. Opioids have shown efficacy in other neuropathic pain conditions [18] [59] [19]. Furthermore, the efficacy of duloxetine in diabetic neuropathy, a condition that has similarities to HIV-SN, may suggest that it is worth investigating [20]. In addition, the efficacy of cannabis in HIV-SN would suggest that cannabinoids with an appropriate therapeutic index when delivered by a mechanism other than smoking might be worthy of investigation [56].

### Conclusions

On the basis of current published evidence, topical capsaicin 8%, smoked cannabis and Nerve Growth Factor have evidence of efficacy in pain associated with HIV-SN. However this is potentially contentious, as a recent larger RCT, currently reported in abstract form only, has suggested this treatment is not superior to placebo [45]. Some commonly recommended analgesics, including opioids, have not been formally studied for the management of painful HIV-SN.

The current evidence base available for the treatment of painful HIV-SN is at odds with the recommendations made by NICE for neuropathic pain management in the non-specialist situation. This indicates the potential dangers of extrapolating efficacy from one neuropathic pain condition to another where efficacy has not been directly assessed. In particular amitriptyline, pregabalin, and gabapentin have been demonstrated to have no superiority to placebo in the treatment of painful HIV-SN.

With an estimated 33 million people living with HIV and more gaining access to ARV every day, the management of HIV-SN associated neuropathic pain is a problem of major global significance. There is an urgent need for the development of effective, evidence based analgesic strategies for this common condition. Gene microarrays have been used to identify novel drug targets [60]. Ongoing evaluation of both novel analgesics and existing untested strategies for HIV-SN is a clear research priority.
